# Evidence of Energetic Optimization during Adaptation Differs for Metabolic, Mechanical, and Perceptual Estimates of Energetic Cost

**DOI:** 10.1038/s41598-017-08147-y

**Published:** 2017-08-09

**Authors:** Natalia Sánchez, Sungwoo Park, James M. Finley

**Affiliations:** Division of Biokinesiology and Physical Therapy, University of Southern California, Los Angeles, CA 90033 USA

## Abstract

The theory that the sensorimotor system minimizes energetic cost during locomotion has long been supported by both computational models and empirical studies. However, it has yet to be determined if the behavior to which people converge when exposed to a novel perturbation during locomotion is also energetically optimal. We address this issue in the context of adaptation to walking on a split-belt treadmill, which can impose a left-right asymmetry in step lengths. In response to this asymmetry, participants gradually adjust their foot placement to adopt steps of equal length. Here, we characterized metabolic, mechanical, and perceptual estimates of energetic cost associated with a range of asymmetries to determine whether symmetry is the energetically optimal strategy for walking on a split-belt treadmill. We found that taking steps of equal length did not minimize metabolic cost or mechanical cost. In addition, perceptual estimates of cost were not sensitive to changes in asymmetry. However, symmetry was identified as the optimal strategy when energetic cost was estimated from a composite metric that combined both metabolic and mechanical costs. These results suggest that adaptation may arise from optimization of a composite estimate of effort derived from feedback about the interaction between the body and environment.

## Introduction

The field of motor control has long aimed to identify the principles underlying how the central nervous system selects from a broad set of feasible behaviors to perform a given motor task. These principles have often been cast in the framework of optimal control theory, which suggests that the sensorimotor system selects behaviors through a continuous optimization of a cost function^1–3^. In the context of locomotion, minimization of energetic cost is one of the most common optimization criteria used to predict behavior. Computational models which use energetic minimization as a behavior selection criterion have successfully reproduced kinematics and patterns of muscle activation during human locomotion^4, 5^. In addition, experiments during steady state walking have shown that self-selected spatiotemporal variables such as step width^6^, step frequency^7–12^, and step time^13^ are those that minimize energetic cost. However, it has yet to be determined whether the behavior that people select when adjusting to novel biomechanical demands during walking is shaped by energetic optimization.

The process of adjusting a well-practiced movement pattern (e.g. walking) when exposed to a perturbation or novel demands is known as motor adaptation^14^. During walking, adaptation often occurs when one is exposed to challenging surfaces such as ice or sand^15, 16^, and when compensating for extrinsic (tripping, slipping, adding loads^17–20^) or intrinsic perturbations (pain or injury, anatomical changes during growth^21–23^). The adaptation process is often explored in the field of motor control through use of novel perturbations such as prism goggles^24^, robotic force fields^25, 26^, visuomotor rotations^27, 28^, and split-belt treadmills^29–31^. In the split-belt adaptation paradigm, participants walk on a treadmill with two separate belts which can be independently controlled to move at different speeds. When exposed to walking on a split-belt treadmill, participants will initially walk with an asymmetric gait characterized by longer steps with the leg on the slow belt and shorter steps with the leg on the fast leg^30, 32, 33^. Over the course of multiple minutes, this asymmetry is reduced, until the difference between the fast and slow step lengths converges toward zero. This adaptation of step length occurs simultaneously with a reduction in both metabolic cost and muscle activity^32^. Although these reductions are consistent with the idea that adaptation results from energetic optimization, they alone are not sufficient to determine whether taking steps of equal length is indeed the energetically optimal strategy.

The general question of whether motor adaptation is driven by energetic optimization has been explored in a few previous studies, however the results have been conflicting. Some studies have reported that adapting to novel dynamics is associated with a reduction in metabolic cost during reaching^34–36^ and minimization of kinematic error and effort during walking^37^. Another study concluded that task performance is prioritized over energetic optimization, such that energetic optimization only occurs after performance has been maximized^38^. In contrast, other studies have concluded that minimization of energy is not an important factor in determining how the central nervous system adapts to novel task dynamics^39–42^. These latter studies have shown that participants do not choose movement patterns that minimize energy, measured as either mechanical work or muscle co-activation, but instead prefer behaviors that are habitual, such as choosing to reach in a straight over a curved trajectory^39–41^, or selecting behaviors associated with recent motor memories^42^. Additionally, studies often weight conclusions drawn from measures of muscle activity, joint mechanics or metabolic cost equally, but it remains to be seen if energetic estimates from multiple sources are comparable or whether the use of a composite estimate of energetic cost can better explain motor behavior.

The concept of energetic cost can be defined at multiple levels of analysis as either metabolic cost^34^, mechanical cost^39, 40^, muscle activation^37, 41, 42^ or perceived effort^43^. For example, mechanical cost can be determined as the sum of the kinetic and potential energy for all limb segments^44^, as the magnitude of positive and negative work performed on the center of mass^7, 11, 45^, or as the amount of braking and propulsion generated to modulate walking speed^46^ and step length^47^. Metabolic cost, measured using indirect calorimetry, can be used to calculate the body’s rate of energy use (metabolic power)^6, 48^ to synthesize ATP. Finally, energetic cost has also been represented by perceived effort, an individual’s perception of the exertion involved in performing a specific task^49^. Thus, determining the energetically optimal behavior can be highly dependent on the method used to quantify energetic cost. Moreover, these metrics may not co-vary in predictable patterns. For example, dissociations between mechanical power output and perceived effort^50^, between perceived effort and oxygen consumption^51^, and between mechanical and metabolic power^13^ have each been reported in the literature. Therefore, quantification of only one metric of energetic cost likely provides an incomplete characterization of energetic optimization processes underlying motor adaptation.

Here, we determined whether adaptation to novel dynamics during locomotion is consistent with the hypothesis that our walking patterns are selected via energetic optimization. We tested the hypothesis that walking with steps of equal length is the most energetically optimal behavior for walking on a split-belt treadmill by mapping the relationship between step length asymmetry and multiple metrics of energetic cost (metabolic cost, perceived effort and lower extremity mechanics). Contrary to our hypothesis, we found that taking steps of equal length was not the metabolically optimal solution for walking on a split-belt treadmill. However, taking steps of equal length did minimize a simple cost function that combined measures of lower extremity mechanics and metabolic cost. These results demonstrate that online minimization of a single energetic cost metric is not the objective of locomotor adaptation. Instead, it is possible that the goal of adaptation is to minimize composite estimates of energetic cost derived from multisensory feedback about the body’s physiological state and the mechanical interaction between the body and environment.

## Results

The purpose of this study was to determine whether the energetic optimization hypothesis is a valid framework to explain how individuals adapt to imposed asymmetries during locomotion. To this end we mapped the relationship between multiple estimates of energetic cost and step length asymmetry during walking on a split treadmill (Bertec, Fully Instrumented Dual-Belt Treadmill). A total of thirty healthy individuals (26 +/− 5 years old, 16 female) participated in this study.

**Figure 1:**
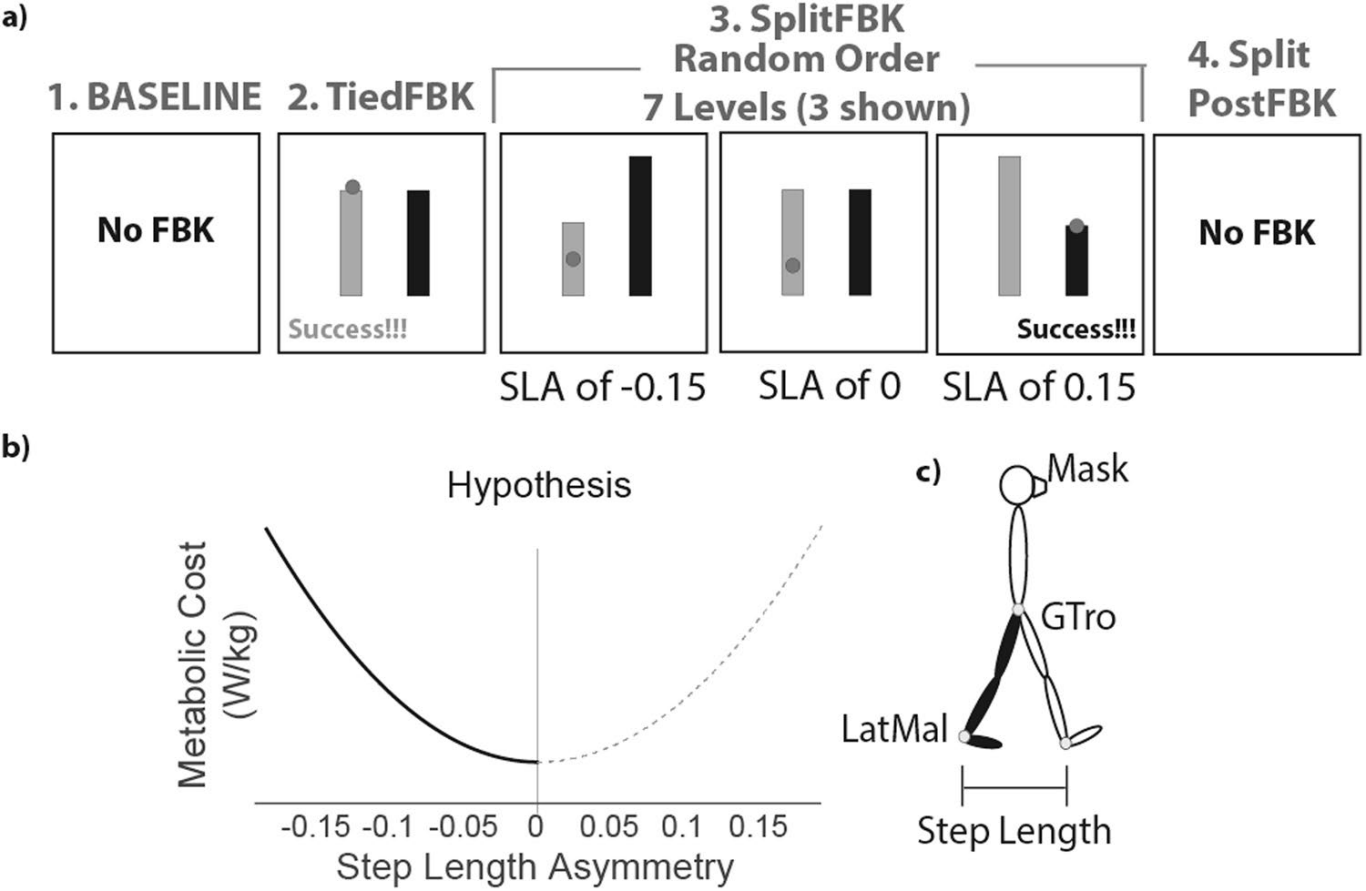
Experimental protocol. (**a**) Visual feedback for all experimental conditions, only three of seven trials of the step length asymmetry (SLA) used during SplitFBK are shown. The lateral malleoli markers were projected on the screen in real time during the swing phase to enable participants to achieve the desired step lengths. A “Success!!!” message appeared on the screen when foot strike occurred within two standard deviations of the desired target. (**b**) Hypothesized relationship between metabolic cost and step length asymmetry. Step length asymmetry values shown on the x-axis were those used as our targets in the SplitFBK trials. The dashed line indicates asymmetries not explored during natural adaptation. (**c**) Schematic of the experimental setup, with the mask used to measure metabolic cost and marker locations for measurement of step lengths. LatMal: Lateral malleolus and GTro: greater trochanter.

Participants walked in three different conditions (Fig. 1a): (1) a 5-minute BASELINE period at 1.0 m/s where we measured average, self-selected step lengths and step length variability; (2) a 5-minute period walking at 1.0 m/s with visual feedback of their self-selected step lengths (TiedFBK); (3) seven, 5-minute walking periods with the left and right belts moving at 1.5 m/s and 0.5 m/s respectively (SplitFBK); and (4) a PostFBK period where participants performed an 8-minute adaptation trial on the split-belt treadmill with no visual feedback. Participants sat and rested for 3 minutes between all trials. Consistent with previous studies^30, 52^, participants were instructed to lightly touch a handrail placed in front of them to aid balance and prevent drift on the treadmill.

Visual feedback of the real-time position of infrared-emitting markers placed on the ankle and the target step lengths were projected onto a large monitor to aid participants in achieving the desired level of step length asymmetry (Fig. 1a). For the TiedFBK condition, participants matched their step lengths to the average step lengths measured during the BASELINE condition to measure the energetic cost increment due to walking with the added dual-task of accurate foot placement^6^. For the SplitFBK trials, step length asymmetry was defined as the normalized difference between their fast and slow step lengths (Equation 1, Methods). Participants were instructed to match the target step lengths on the right and left as soon as they began walking on the split-belt treadmill. Thus, they were not allowed to implicitly adapt to the split-belt perturbation but were explicitly clamped at specific step length asymmetries. All participants walked with target asymmetries of zero, +/−0.05, +/−0.10 and +/−0.15 with negative values corresponding to longer steps with the slow (right) leg and positive values corresponding to longer steps with the fast (left) leg. The negative values were selected to be consistent with the ranges that typically occur during locomotor adaptation^30, 32, 53^, and the positive values were selected to provide a uniform distribution of asymmetries around zero (Fig. 1b).

### Voluntary modification of step length asymmetry

**Figure 2:**
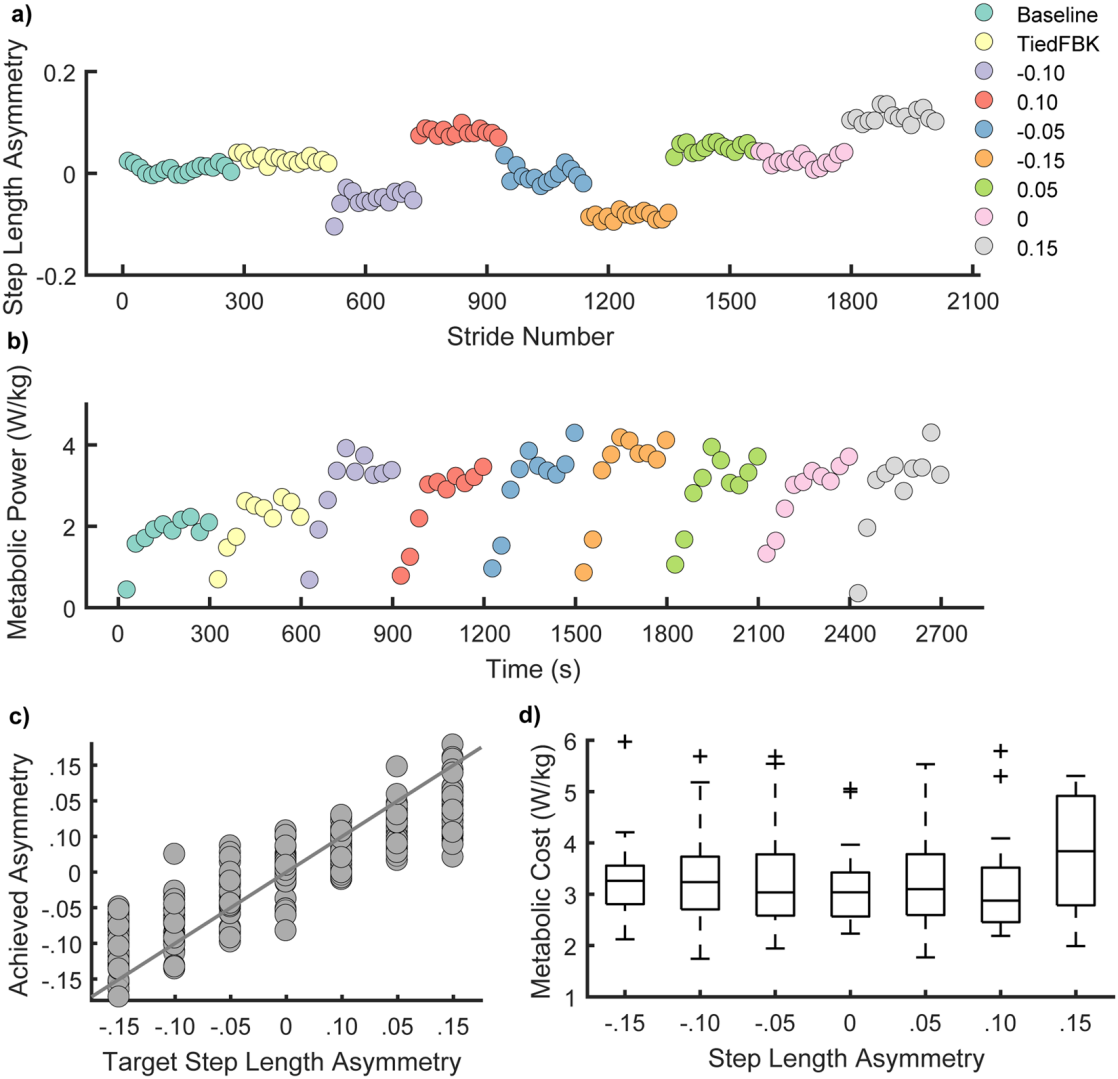
Metabolic cost and step length asymmetry. (**a**) Raw data for a representative participant for step length asymmetry. Each data point represents the average asymmetry during a 15-stride interval. The target asymmetries for this participant were presented in the following order: −0.10, 0.10, −0.05, −0.15, 0.05, 0 and 0.15. (**b**) Raw metabolic cost data for the representative participant in (**a**). Each data point corresponds to the average metabolic power for a 30s interval. (**c**) Target step length asymmetry vs. achieved step length asymmetry for all participants (N = 30). Accurate performance would follow the gray the line. Participants typically undershot the targets for the end ranges (+/−0.15) due to the difficulty of the task. (**d**) Distributions of metabolic cost associated with each level of achieved asymmetry.

Overall, there was no significant difference between the mean step length asymmetries during BASELINE and TiedFBK (BASELINE: 0.0031 +/−0.016, TiedFBK: 0.0039 +/−0.023, paired-t test p = 0.71), indicating that participants were able to maintain their natural step lengths using visual feedback. During subsequent trials, participants systematically manipulated their step length asymmetry while walking on the split-belt treadmill (Fig. 2a and c). Participants rapidly updated their step length asymmetry using visual feedback and maintained this asymmetry for the duration of each five-minute trial.

### Effects of voluntary modification of foot placement on energetic cost

**Figure 3:**
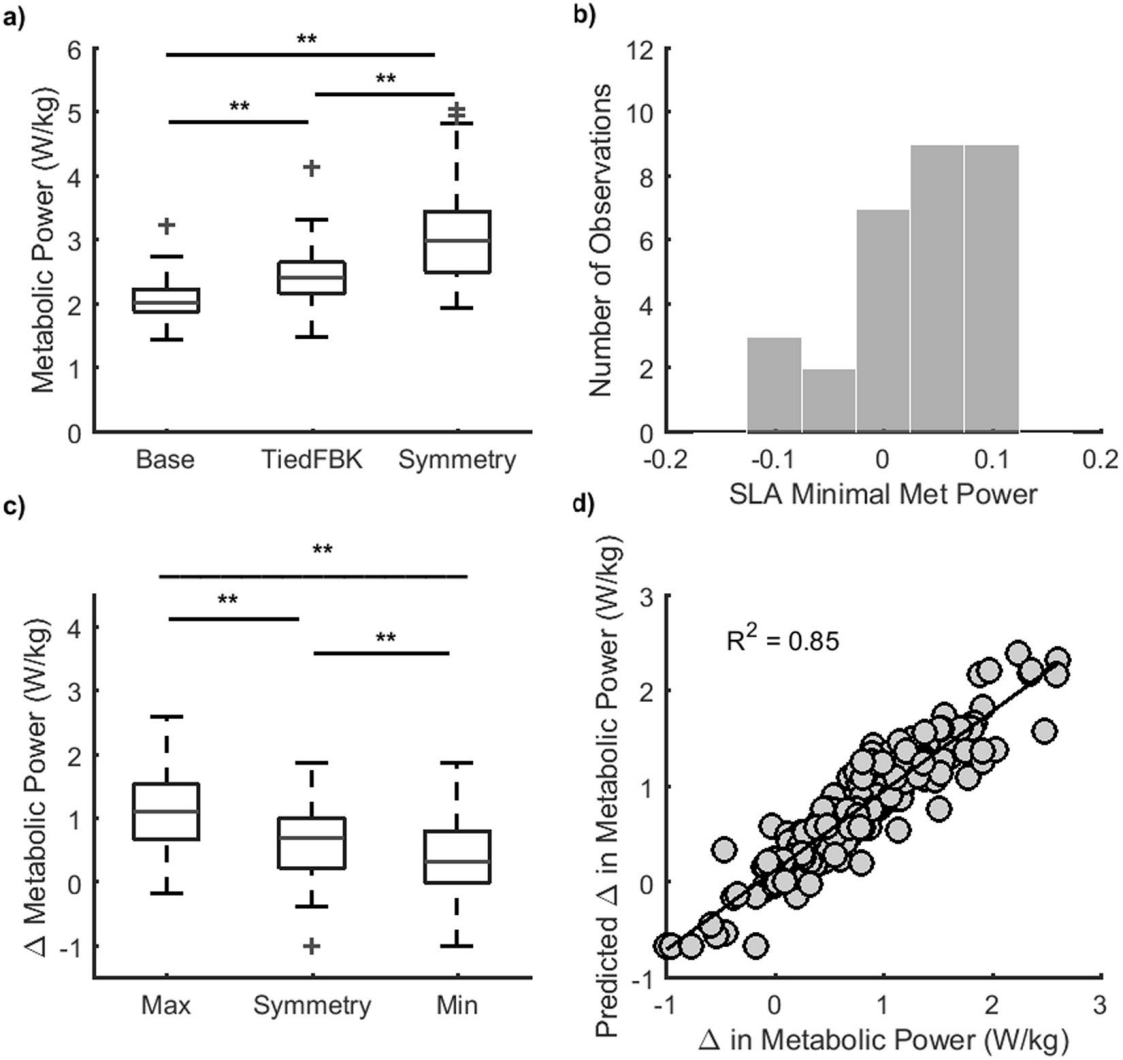
Associations between step length asymmetry and metabolic cost. (**a**) Metabolic cost measured during baseline walking, walking with feedback (TiedFBK) and symmetric walking with belts split (SplitFBK at 0% asymmetry). Significant increases in metabolic cost from BASELINE were measured for all conditions (p < 0.001). (**b**) Histogram of step length asymmetries (SLA) associated with the minimum metabolic cost for all participants. For the distribution of step length asymmetries that produced the minimum cost, values were biased towards asymmetries greater than zero (longer steps on the fast belt). (**c**) Maximum and minimum metabolic cost and metabolic cost during symmetry in the SplitFBK conditions. Here, metabolic cost is expressed as an increase from the TiedFBK conditions to account for the cost of precise foot placement using visual feedback. Significant differences between the maximal, minimal and symmetric metabolic costs were measured at the p < 0.001 level. (**d**) Predicted metabolic cost derived from the linear mixed effects model that tested for associations between asymmetry, direction of asymmetry and metabolic cost (Equation 3 in Methods).

The voluntary control of step lengths through use of visual feedback resulted in modification of metabolic power (Fig. 2b) and lower extremity mechanics. Metabolic power was assessed using measures of oxygen consumption and carbon dioxide production, determined from indirect calorimetry. Across participants, precise foot placement in the TiedFBK condition resulted in an increase in metabolic power of 18% +/−13% above BASELINE values (Fig. 3a, p < 0.001). In addition, propulsive impulses increased by 20% +/− 15% and 20% +/− 14% on the left and right side, respectively (both p < 0.001), while braking impulses increased by 12% +/− 17% and 10% +/−17% on the left and right sides, respectively (both p < 0.001). Given the metabolic and mechanical effects of the visual feedback, energetic estimates for the SplitFBK conditions are subsequently expressed as a change from the TiedFBK condition.

Next, we examined the effect of having the belts move at different speeds on the metabolic power associated with a step length asymmetry of zero. Compared to the TiedFBK condition, walking with equal step lengths in the SplitFBK condition generated an increase in metabolic power of 28% +/− 24% (p < 0.001, Fig. 3a). This indicates that walking with the belts moving at different speeds results in a substantial increase in metabolic power even if the level of asymmetry is the same as baseline walking. Importantly, the metabolic power measured when participants used visual feedback to maintain a symmetric walking pattern (3.14 ± 0.80 W/kg) was comparable to the metabolic power measured in a previous study when symmetry was achieved through implicit adaptation processes (3.05 ± 0.24 W/kg^32^).

Manipulation of step length asymmetry during the SplitFBK trials resulted in a large variation in metabolic power across trials (Fig. 2b and d, Supplementary Fig. S1). Based on our hypothesis, we expected that the step length asymmetry associated with the lowest metabolic power would be normally distributed around zero, consistent with symmetry being the energetically optimal solution for walking on a split-belt treadmill. Kolmogorov-Smirnov tests rejected the null hypothesis that the asymmetries associated with the lowest power were normally distributed (Fig. 3b, p < 0.001). The sign test also rejected the null hypothesis that the distribution’s median was equal to zero (p = 0.016). The distribution of step length asymmetries associated with the minimum metabolic power was biased towards positive values with a median of 0.046, an interquartile range of 0.080 and skewness of −0.796, indicating an asymmetric distribution with greater spread to the left of the mean.

Figure 3c illustrates the metabolic power increment, expressed as a difference from the TiedFBK condition, for the most costly and least costly tasks as well as the metabolic power increment for symmetric walking. The minimum metabolic power increment relative to the TiedFBK condition was 0.40 +/− 0.59 W/kg. The power increment associated with taking equal step lengths was 0.66 +/− 0.61 W/kg, and the highest metabolic power increment across all asymmetries was 1.15 +/− 0.67 W/kg. These values were all significantly different from one another (repeated measures ANOVA, all p < 0.001) and together demonstrate that the optimal metabolic cost was significantly less than the cost associated with symmetry, indicating that symmetry is not metabolically optimal.

To further explore associations between metabolic power and asymmetry, we fit a linear mixed effect model to the metabolic power data (Equation 3 in Methods) and obtained the simplest model that best represented the data, based on a likelihood ratio test. Significant fixed effects were found for *Asymmetry* magnitude (p < 0.001) and the interaction between *Leg* and *Asymmetry* (p = 0.048), indicating that the effect of asymmetry depended on which leg took the longer step (Table S1). Specifically, metabolic power was greater when the slow leg took a longer step than when the fast leg took the longer step. The variance of the random intercept across participants was significantly different from zero which indicates that it was necessary to model the variation in metabolic power due to random differences between participants. We calculated effect sizes using Cohen’s *d* ^54^ and obtained a value of 0.62 for the independent variable *Asymmetry*, and 0.36 for the interaction term i.e., medium effect sizes. This model had an R^2^ of 0.85 (Fig. 3d).

Previous research has shown that changes in cadence are associated with modifications in the metabolic cost of walking^8^ which could have influenced our results. Therefore, we used mixed effect models to determine whether stride time varied with changes in step length asymmetry and if these changes explained significant variance in metabolic cost. Significant effects of step length asymmetry on stride time were observed (p = 0.002, Table S2), but there were no significant effects of stride time on metabolic cost (p = 0.18, Table S3, Fig. S4). Therefore, the changes in metabolic cost measured were not driven by changes in cadence.

### Associations between step length asymmetry and lower extremity mechanics

**Figure 4:**
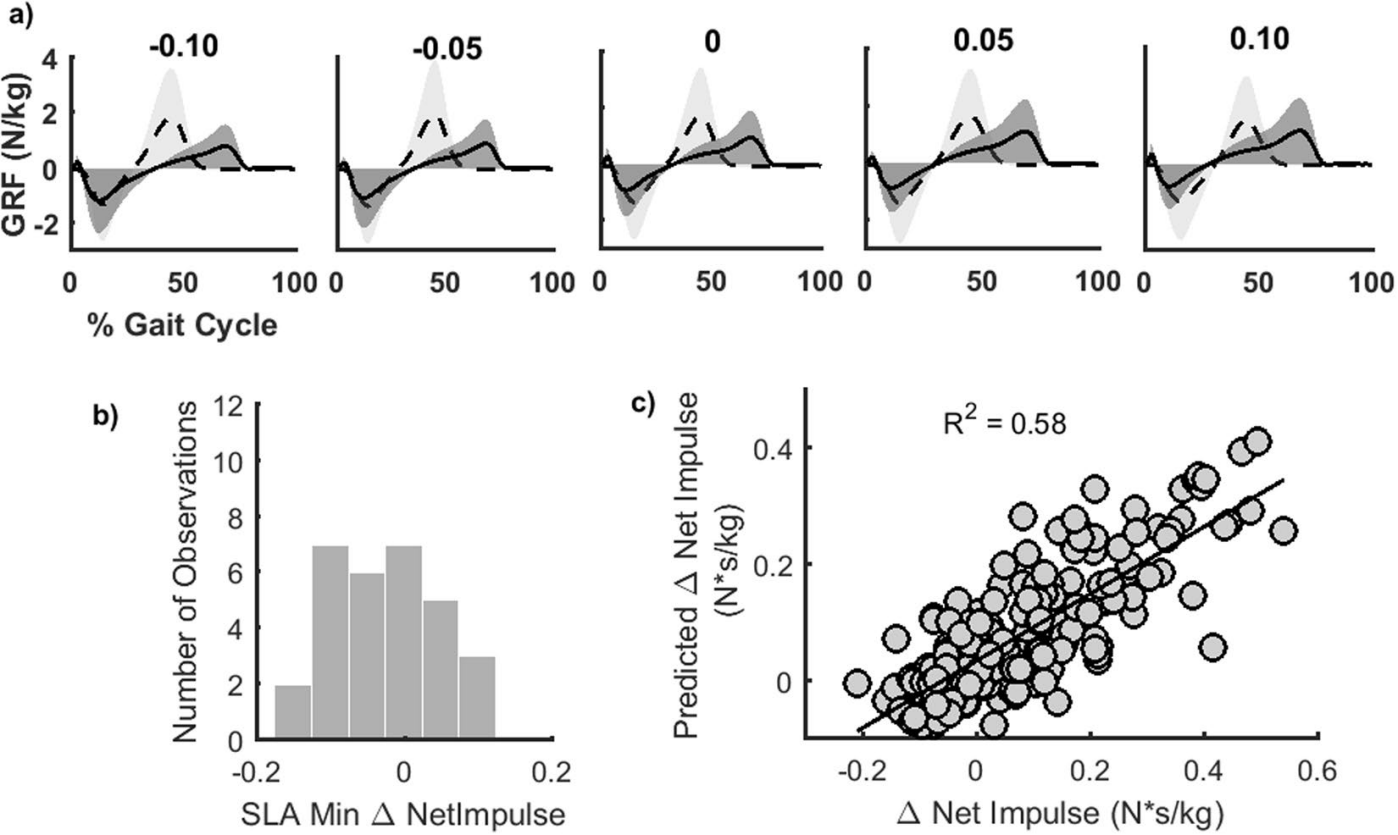
Associations between braking and propulsive impulses and asymmetry. (**a**) Fore-aft ground reaction forces for each achieved step length asymmetry for the fast (dashed) and slow (solid) leg. Data are pooled by achieved asymmetry, not by target asymmetry. The braking impulse was defined as the area under the negative portion of the leading limb’s fore-aft ground reaction curve during the first half of the gait cycle. Similarly, the propulsive impulse was computed as the area under the curve for the positive portion of the trailing limb’s fore-aft ground reaction force in the second half of the gait cycle. (**b**) Histogram of step length asymmetries associated with the minimum net impulse. (**c**) Plot of measured vs. predicted net impulses using the linear mixed effects model defined in Equation 4.

To examine the possibility that energetic optimization occurs in the domain of lower extremity mechanics, we explored the distribution of step length asymmetries that minimized the net impulse across limbs. Net impulses were baseline corrected to those measured in the TiedFBK condition to control for the effects of the accurate foot placement due to the added visual feedback on lower extremity mechanics. Propulsive and braking impulses corresponded to the area under the curve for the positive and negatives values of the fore-aft ground reaction force, respectively. Across asymmetries there were systematic variations in fore-aft ground reaction forces and resulting braking and propulsive impulses for each limb (Fig. 4a, Fig. S2). Because both braking and propulsive impulses require muscle contraction and contribute to total energetic cost, we summed the magnitude of the braking and propulsive impulses for the fast and slow legs to obtain the net impulse. Kolmogorov-Smirnov tests rejected the null hypothesis that the step length asymmetries associated with the net impulse minima were normally distributed (p < 0.001). However, the sign test failed to reject the null hypothesis that the median of the distribution of step length asymmetries that minimized net impulse was equal to zero (p = 0.58). The step length asymmetry associated with the lowest net impulse increment had a median of −0.03, an interquartile range of 0.12, and a skewness of 0.21 (Fig. 4b). Although the median was not significantly different from zero, the width of the interquartile range indicates that symmetry was only optimal for a subset of participants, and in fact, optimal net impulses across participants were observed across the entire range of step length asymmetries tested except for +0.15.

We further explored the relationship between the net impulse and step length asymmetry using linear mixed effects models. We found that the net impulse was associated with *Asymmetry* magnitude (p = 0.03) and the interaction between *Asymmetry* and *Leg* (p = 0.02). This indicates that impulse magnitude increased more for positive asymmetries than negative asymmetries. Based on the AIC, the model required the inclusion of a random intercept for each participant. The model explained 58% of the variability in impulses (Table S4, Fig. 4c). Net impulses were also strongly associated with metabolic cost (p = 0.03, Table S5).

### Associations between step length asymmetry and composite estimates of energetic cost

**Figure 5:**
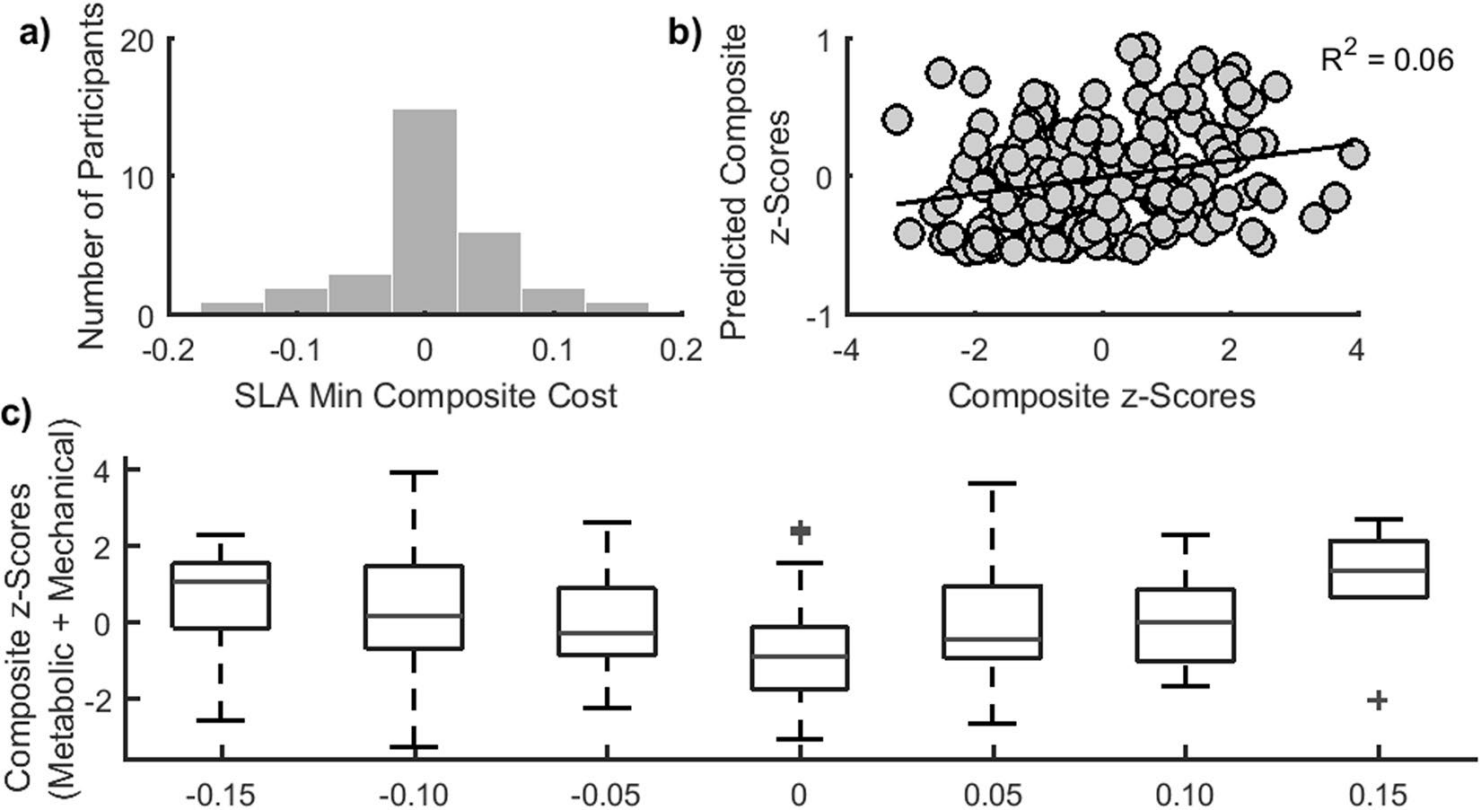
Associations between a composite estimate of energetic cost and asymmetry. (**a**) Histogram of step length asymmetries associated with the minimum composite cost. (**b**) Plot of measured vs. predicted composite costs defined in Equation 5. (**c**) Distributions of composite costs associated with each level of achieved asymmetry. These results match the hypothesized relationship presented in Fig. 1b.

We next examined whether a combination of costs could explain why participants converged to symmetry during locomotor adaptation. Given that each of our energetic metrics measure different phenomena and are expressed in different units, we derived z-scores for each metric and summed the z-scores to obtain a composite estimate of energetic cost. We obtained a metabolic and mechanical composite z-score for all participants (Fig. S3). The Kolmogorov-Smirnov test rejected the null hypothesis that the step length asymmetries associated with the minimal composite cost were normally distributed (p < 0.001). However, the sign test failed to reject the null hypothesis that the median of the distribution of step length asymmetries that minimized the composite cost was equal to zero (p = 0.58). The distribution had a median of 0.008, a skewness of −0.45 and an interquartile range of 0.05 (Fig. 5), indicating that the step length asymmetry associated with the minimal composite cost was close to symmetry in 75% of participants. Linear mixed effects models to test associations between the composite cost, *Leg*, and *Asymmetry* (Equation 5) only included an intercept and *Asymmetry* as significant predictors (both p < 0.01, Table S6). Thus, any asymmetry different from zero would lead to an increase in the composite cost. These results corroborate our initial hypothesis that symmetry is energetically optimal during split belt walking, but only when energetic cost is quantified using a composite estimate.

### Perceived Exertion

**Figure 6:**
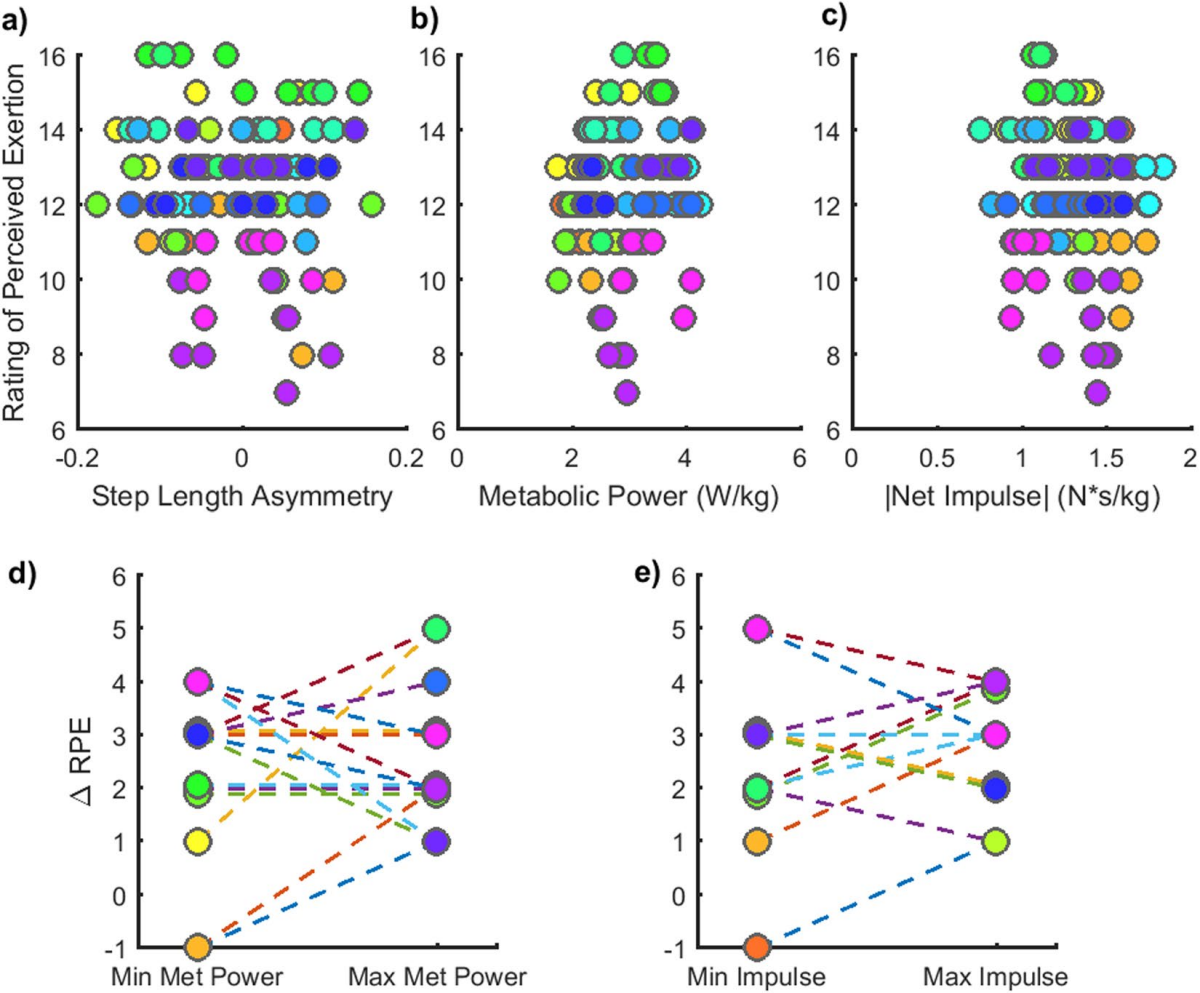
Ratings of perceived exertion (RPE). (**a**) RPE associated with each achieved step length asymmetry. (**b**) RPE as a function of the measured metabolic power. (**c**) RPE as a function of the measured net impulse. (**d**) RPE for the asymmetries associated with minimum (left) and maximum (right) metabolic power. Only five participants reported an increase in perceived exertion for the condition where the maximum metabolic cost was observed relative to the condition where the minimum cost was observed. (**d**) RPE for the asymmetries associated with minimum (left) and maximum (right) net impulse. No explicit awareness of the mechanical effort required was reported.

For a subset of 15 participants, we assessed self-reported perceptions of effort using the Rating of Perceived Exertion (RPE) scale^55^ after each 5-minute walking trial. No visible relationship between RPE and asymmetry (p = 0.44), between RPE and metabolic power (p = 0.43) or between RPE and net impulses (p = 0.48) was observed (Fig. 6a–c). Surprisingly, there were many cases where participants indicated either the same or lower levels of perceived effort for trials that corresponded to their maximum energetic cost relative to trials where the minimum cost was observed (Fig. 6d,e). Because participants often indicated their lowest RPE for multiple trials across a range of asymmetries, it was not possible to discern the level of asymmetry that corresponded to their lowest RPE. Overall, our assessment of RPE revealed that participants had no explicit, systematic awareness of the differences in metabolic or mechanical cost associated with the levels of step length asymmetry explored in this study.

We also obtained a composite score that included perceptual metrics of effort for the subset of 15 participants in which perceived effort was quantified. For the composite cost using all three metrics of energetics (metabolic, mechanical and perceptual), the distribution of asymmetries associated with the minimal composite was flat (Fig. S5). The flatness of the distribution may be due to the fact that 15 observations may not effectively determine the distribution of optimal step length asymmetries.

### Effects of prior exposure to energetically optimal solutions on split-belt adaptation

**Figure 7:**
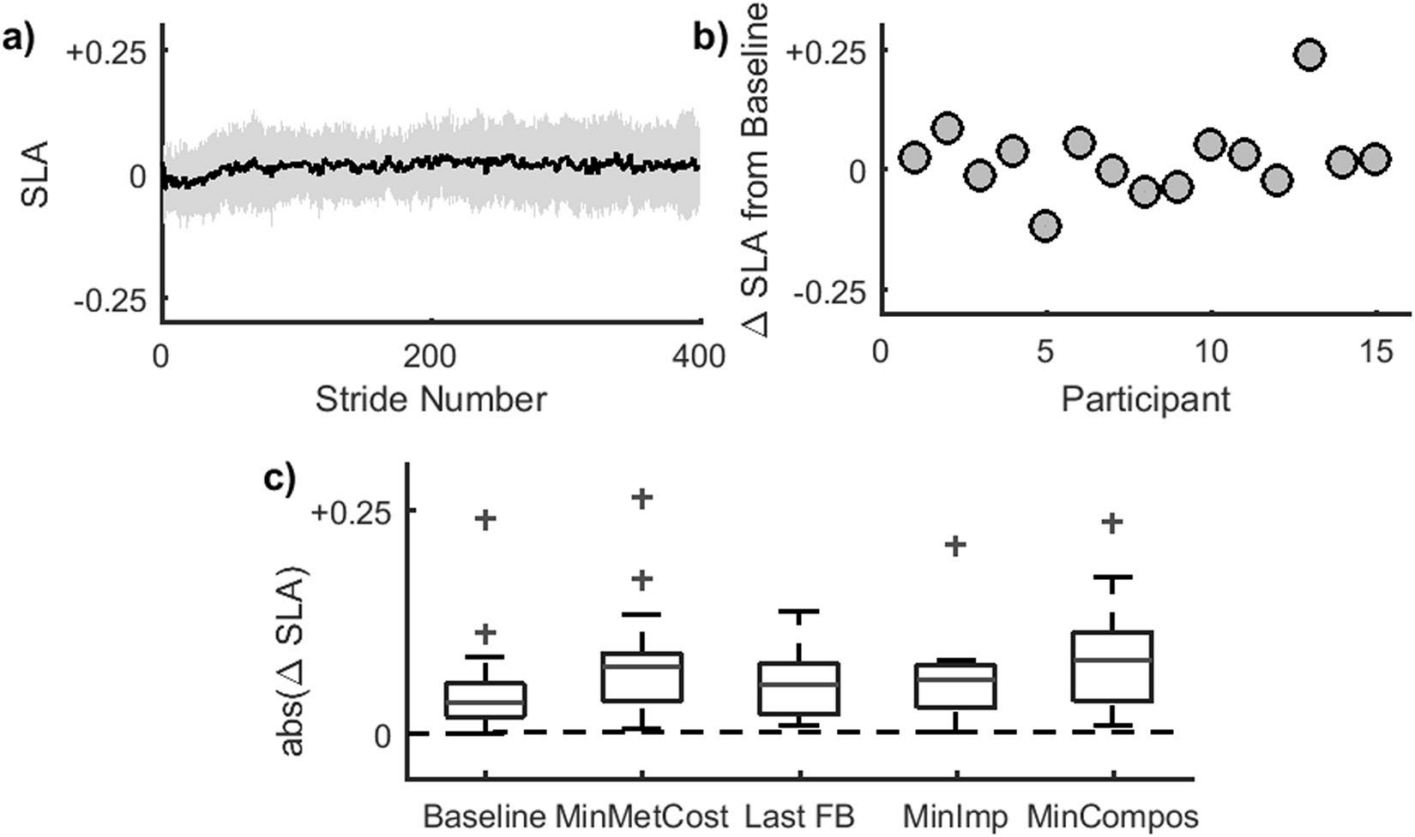
Step length asymmetry during PostFBK. (**a**) Average step length asymmetry (SLA) adaptation curve for the PostFBK trial for 15 participants. The shaded area surrounding the curve represents the standard error. (**b**) Difference between the final step length asymmetry during PostFBK relative to the baseline asymmetry for each participant. (**c**) Magnitude of the difference in step length asymmetry during the PostFBK trial compared to baseline (Base), the asymmetry associated with the minimum metabolic cost (MinMetCost), the last SplitFBK trial (LastFB), the asymmetry associated with the minimal impulse (MinImp) and the asymmetry associated with the minimal composite cost (MinCompos). The distribution of step length asymmetries observed at the end of the PostFBK trial was closest to each participant’s baseline asymmetry.

Fifteen participants also completed an 8 minute period of split-belt adaptation with no visual feedback after all SplitFBK trials (PostFBK, Fig. 7a). This allowed us to determine whether exploration of the energetic cost landscape led participants to converge to a step length asymmetry that minimized metabolic cost, as has been demonstrated in other forms of locomotor learning^56^. For this analysis, we computed the difference between the final level of asymmetry during the PostFBK condition and the asymmetry observed during (a) BASELINE walking, (b) the conditions in which the lowest costs were observed (metabolic, mechanical and composite), and (c) the asymmetry observed in the final SplitFBK trial. We found that the difference in asymmetry between PostFBK and BASELINE was significantly smaller than the difference between PostFBK and all other conditions (repeated measures ANOVA p < 0.05), (Fig. 7c). The step length asymmetry to which participants converged during PostFBK was associated only with their baseline asymmetry (p = 0.017) and was not at all associated with the energetically optimal strategy as measured by metabolic cost (p = 0.77), mechanical cost (p = 0.95), mechanical and metabolic composite cost (p = 0.35), the composite using all three metrics of energetic cost (p = 0.31), or the asymmetry generated during the final SplitFBK trial (p = 0.12). The outliers in Fig. 7c come from one participant who converged to an asymmetry of 0.26 in the absence of feedback. This individual was biased toward the large positive asymmetry experienced in the final SplitFBK trial. Removing this participant from the analysis did not change results. These results indicate that prior exposure to more metabolically optimal strategies does not alter participants’ self-selected walking patterns on the split-belt treadmill, in contrast to previous work^56^.

## Discussion

Energetic optimization has long been proposed as a method by which we select locomotor patterns^6–13^. However, whether gait features are selected to minimize energetic cost in the context of locomotor adaptation had yet to be determined. To test the hypothesis that symmetry is the energetically optimal solution for adapting to walking on a split-belt treadmill, we mapped the energetic cost landscape associated with different levels of step length asymmetry. Surprisingly, and contrary to our hypothesis, we found that symmetry was not the energetically optimal solution to walking on a split-belt treadmill when energetics was assessed using metabolic cost. Instead, our results indicate that positive step length asymmetries (longer steps on the fast belt and shorter steps on the slow belt) optimized metabolic cost below the cost of symmetry, despite the fact that these asymmetries are rarely, if ever, explored during adaptation. In contrast, mechanical measures of energetic cost were not sensitive to changes in asymmetry. This observation that multiple estimates of energetic cost produced conflicting support for the energy minimization hypothesis suggests that assessments of single metrics of energetic cost may not be appropriate for assessing the role of effort minimization in the study of motor control. In fact, here we show that taking steps of equal length appeared to be optimal for the majority of our participants when energetic cost was estimated using a composite metric combining metabolic and mechanical costs.

A potential explanation for why equal step lengths are selected during split-belt walking despite the metabolic cost penalty is that the motor system may be more concerned with or better able to estimate a composite cost that simultaneously reflects the dynamics of the interaction between the body and environment. Because we did not observe associations between ratings of perceived effort and step length asymmetry, this suggests that variations in cost due to asymmetry did not reach conscious awareness and may instead contribute to optimization of asymmetry through implicit, sub-conscious processes.

We initially expected results from estimates of energetic cost based on expired gas analysis and lower extremity mechanics to show reasonable agreement such that each metric would be minimized at the same step length asymmetry. Although this is not what we observed, it is possible that other metrics like EMG^57^, joint moments^45^ or work performed on the center of mass^11^ may be optimized for similar asymmetries as metabolic cost. A close association between metabolic and mechanical estimates of energetic cost has been demonstrated in previous studies where metabolic cost was assessed in conjunction with the mechanical cost of transport^6, 11^. However, this work demonstrated that the relationship between metabolic and mechanical cost diverged for habitual behaviors such that the actual metabolic cost was lower than what would be predicted based on mechanics. This divergence could result, for example, from reductions in agonist/antagonist co-contraction that would not be captured through mechanical estimates. A potentially interesting area of exploration for future studies would be to develop a paradigm where mechanical and metabolic costs can be manipulated independently of one another to further elucidate potential energetic optimization principles in motor control during habitual and novel behaviors.

The results from our PostFBK trial contrast a recent study which concluded that participants adopt gait features that corresponded to recently-experienced, metabolically optimal strategies^56^. Here, we observed that when visual feedback was removed and participants were allowed to freely choose their walking pattern, they did not converge to the recently experienced, metabolically optimal strategy, but instead converged to patterns strongly associated with their own baseline level of symmetry. Importantly, the absolute metabolic cost savings for the energetically optimal strategy in our study were comparable to those reported previously^56^. One potential explanation for this discrepancy is that there may have been a strong association between mechanical energetics (i.e. ground reaction force impulses) and metabolic cost in the prior study which would prevent one from determining which of the two variables are most important to the central nervous system. Despite our findings that symmetry was optimal when energetics was quantified using a composite cost, the asymmetry participants converged to in the absence of feedback was not associated with the optimal composite cost. This may be due to the fact that for these analyses we only used a subset of our study sample.

Although energetic cost and effort can be characterized using a number of objective (metabolic cost, joint moments, center of mass work, etc.) and subjective (perceived effort) assessments, our understanding of how effort is represented by the nervous system and how this representation influences action selection may benefit from the use of composite metrics that account for the differential contribution of these signals to central representations of effort. The use of composite scores has been proposed in the domain of cognitive science^58^ where one is interested in the value of some underlying psychological construct (e.g. intelligence), but lacks a direct measure of its true value. A solution to this problem is to combine multiple, imperfect measurements thought to correlate with the underlying construct and create a composite score using these measures^59^. This is, in principle, similar to improvements in sensory estimation resulting from the weighting of estimates from multiple sensory modalities^60, 61^. Because a generalized, central representation of effort is likely to be multidimensional and influenced by both cognitive and physical demands^62^, the study of effort in motor control may be best served by the standard use of composite metrics based on multiple measures in each sub-domain.

Although this study focused on energetic optimization, it is also plausible that adaptation is driven by other objectives such as balance maintenance, minimizing injury risk, reducing sensory prediction errors, or even optimization of a multifactorial cost function. Recent research has shown that adaptation to maintain balance during perturbations of whole body movements, such as a squat to stand task, occur over a much faster timescale than what is typically observed during other motor adaptation studies such as reaching, because of the potentially catastrophic consequences of a fall^63^. With respect to the current study, it is possible that step length symmetry is preferred because it is a safer strategy given that prolonged use of an asymmetric walking pattern could increase the risk of injury due to overuse of one limb. It has also been demonstrated that learning, as measured by the presence of an aftereffect, can occur without explicit changes in behavior during adaptation, which suggests that learning can be driven through minimization of sensory prediction errors^64^. Lastly, symmetric walking patterns could be preferred because taking steps of equal length has been reinforced over a lifetime and may be the nervous system’s preferred strategy even if it requires one to use unequal step times. In line with this idea, previous studies have observed that habitual behaviors, or those that have been practiced over a lifetime, are often selected over those that may further reduce energetic cost^39–42^. A key challenge in identifying central principles of action selection is that multiple hypothesized processes may yield similar behaviors in a given experimental paradigm. As a result, there still remains a need to develop innovative approaches capable of distinguishing between competing hypotheses.

One of the limitations of our study is that there was considerable variability in performance across participants, with only seven participants successfully achieving all seven levels of step length asymmetry, while all other individuals undershot the end ranges (Fig. 2c and d). In order to provide a more complete mapping of the landscape relating step length asymmetry and associated costs, future studies should increase the reward provided to participants to better encourage accurate performance^65^ or repeat trials where the target was not achieved. Another possible limitation is that participants held on to a handrail during the study, to prevent drifting on the treadmill. Light touch to a fixed surface during treadmill walking can aid stability^66^. Given that we reduced the requirement for active control of stability, which is another potential cost function to be optimized, we believe that our paradigm would be well-suited to promote energetic optimization, given that balance maintenance was not a concern. The use of visual feedback to explicitly control step length asymmetry may also not reflect the same processes involved in more implicit adaptation. Whether or not this is true, our measures of metabolic cost were comparable to those observed during implicit selection of step lengths during adaptation^32^. This suggests that the change in metabolic cost due to precise foot placement has a negligible influence on metabolic cost during split-belt walking. Lastly, another potential limitation in our study is the use of z-scores to obtain a composite energetic cost. This is primarily evidenced by the low R^2^ value for our model relating *Asymmetry* and the composite cost. Future work should focus on developing systematic approaches for combining estimates of energetic cost from multiple sources. In particular, approaches for creating composite metrics using non-standardized parameters that allow for subject-specific effects may provide a better explanation of observed behavior.

## Conclusion

This study demonstrates that taking steps of equal length does not appear to be the energetically optimal strategy for walking on a split-belt treadmill when energetic cost is captured using single metrics. However, symmetry may be the optimal strategy when cost is assessed using a composite metric of energetics that combines both the mechanical effort necessary to interact with the environment and the physiological cost of adapting to a perturbation. Because motor skill learning is the basis of many therapies aimed at movement rehabilitation and recovery, further understanding of the objectives that drive movement selection by the central nervous system can aid in the development of more effective interventions for the recovery of walking function. Future research should continue to identify the potential hierarchy of goals driving locomotor learning, be it optimization of a single cost, optimization of a weighted sum of multiple costs, or a desire to converge toward features of behavior exhibited in everyday life.

## Methods

Thirty healthy participants completed our study. All experimental procedures were approved by the University of Southern California Institutional Review Board and each participant provided written, informed consent before testing began. All aspects of the study conformed to the principles described in the Declaration of Helsinki.

### Mapping Energetic Cost using Online Feedback of Step Length Asymmetry

We mapped the relationship between multiple estimates of energetic cost (metabolic cost, braking and propulsive impulses, and perceived effort) and step length asymmetry to determine whether symmetry was the optimal behavior for walking on a split-belt treadmill. To achieve the desired level of step length asymmetry, real-time visual feedback of step lengths was provided during a series of 5-minute walking trials. For all feedback conditions, participants viewed the desired step length targets and the real-time location of makers on their ankles via a 52″ screen placed in front of the treadmill. Participants were instructed to match the feedback immediately after the treadmill was started, and therefore participants did not adapt to the treadmill but used explicit feedback to generate specific step lengths during each trial. Ankle location feedback was provided during the swing phase of each leg. The display was controlled by custom software written in Vizard (Worldviz, Santa Barbara, CA). Participants were instructed to step such that foot strike would occur within two standard deviations (as measured during BASELINE) of the target step length asymmetry. A “Success!!!” message appeared on the screen whenever this condition was satisfied. Participants were encouraged to achieve as many “Success!!!” messages as possible (Fig. 1a).

Step length asymmetry was defined as follows:

1 $$Step\,Length\,Asymmetry=\frac{S{L}_{fast}-S{L}_{slow}}{S{L}_{fast}+S{L}_{slow}}$$

Here, step length (SL) is defined as the fore-aft distance between the markers on the lateral malleoli at the time of the respective limb’s footstrike. During all feedback trials we constrained the sum of the step lenghts to be equal to the baseline stride length as follows:

2 $$S{L}_{fast}+S{L}_{fast}=Strid{e}_{baseline}$$

Participants performed SplitFBK trials with target asymmetries of 0, +/−0.05, +/−0.10 and +/−0.15 with negative values corresponding to longer steps with the slow (right) leg and positive values corresponding to longer steps with the fast (left) leg. The order of all SplitFBK trials was randomized to reduce any systematic effects of learning or fatigue. A vital feature of our design was the measurement of positive step length asymmetries. As illustrated in Fig. 1b, positive step length asymmetries are not typically explored during split-belt adaptation. Although it is necessary to characterize this range of asymmetries to gain a full picture of the energetic cost landscape of split-belt walking, no previous studies have done so. Fifteen of the thirty participants also completed a PostFBK trial, which allowed for locomotor adaptation to occur and involved walking without augmented feedback of their step lengths. This trial was used to determine the level of asymmetry they naturally selected when walking on a split-belt treadmill.

### Data Acquisition

Kinematic data for active, infrared markers placed bilaterally on the lateral malleoli and greater trochanters were collected using an 11 camera Qualisys Oqus camera system (QTM, Sweden). Markers on the lateral malleoli were used to measure step lengths^30, 33, 64^ (Fig. 1c). Foot strike and lift-off were estimated from peak anterior and posterior lateral malleoli excursions, respectively^67^. Stride times were defined as the time between successive foot strikes of the same limb. Ground reaction forces were recorded from force plates located under each belt.

Metabolic cost was assessed using expired gas analysis. Expired gas was sampled on a breath-by-breath basis from a mask that covered the participant’s nose and mouth, and the rate of oxygen consumption and carbon dioxide production were measured using a TrueOne^®^ 2400 system (Parvomedics, UT).

### Data processing and analysis

#### Lower Extremity Mechanics

We measured changes in lower extremity mechanics as a function of step length asymmetry to determine if energetic optimization might occur in the domain of mechanical energetics. Fore-aft ground reaction forces were obtained from each force plate (Fig. 4a) to calculate braking and propulsive impulses on the fast and slow belt for each condition. The braking impulse was defined as the area under the negative portion of the leading limb’s fore-aft ground reaction curve during the first half of the gait cycle. This corresponds to the contribution of the leading limb to reduce the forward momentum of the body. Similarly, the propulsive impulse was computed as the area under the curve for the positive portion of the trailing limb’s fore-aft ground reaction force in the second half of the gait cycle. This propulsive impulse corresponds to the contribution of the trailing limb to increase the body’s forward momentum.

#### Metabolic cost

Measures of oxygen consumption and carbon dioxide production were used to compute metabolic power based on a standard equation^48^. The average metabolic power from a standing baseline trial was subtracted from measurements made during all subsequent walking periods to yield net metabolic power. The metabolic power corresponding to each level of asymmetry was calculated as the average net metabolic power measured during the last two, steady state minutes of each trial, which corresponds to approximately the last 100 strides used for the step length asymmetry analyses. To facilitate comparison between participants, all metabolic power data were expressed as a difference from the cost of the TiedFBK condition, to remove the cost resulting from participants’ efforts to attend to the feedback and make precise foot placement. All measures of metabolic power were normalized by body mass.

For 15 participants we assessed self-reported ratings of perceived effort during the SplitFBK trials to test the hypothesis that perceived effort is a reliable proxy for metabolic cost during walking on the treadmill. Perceived effort was assessed using the Borg Rating of Perceived Exertion (RPE) scale^55^. After each 5-minute walking trial, participants were presented with a table containing the RPE Scale, and then indicated the number that best described their perceived level of effort for each trial. Similar to our measures of metabolic cost, the RPE during the SplitFBK trials was expressed as a difference from the RPE for the TiedFBK condition.

### Statistical analyses

All statistical analyses were performed in Matlab version R2015b (Mathworks, Natick, MA, USA). Paired sample t-tests were used to test the null hypothesis that step lengths, metabolic cost, and braking and propulsive impulses were equal in the BASELINE and TiedFBK conditions. We did not expect to observe differences in step length between conditions but did expect that there might be differences in metabolic cost and mechanics due to the precise foot placement requirements of the TiedFBK trial. Repeated measures ANOVAs were run to test differences in the metabolic power between BASELINE, TiedFBK and symmetry conditions. Post-hoc analyses were run using Tukey's honest significant difference criterion.

We then tested the hypothesis that step length asymmetries of zero, i.e., symmetry, corresponded to the minimum metabolic cost. For this analysis, we first identified the level of step length asymmetry that corresponded to each participant’s minimum metabolic cost. Because the asymmetries associated with the minimum metabolic cost did not follow a standard normal distribution based on the Kolmogorov-Smirnov test, the Sign test was used to test the hypothesis that the median of this distribution was zero. To quantify the asymmetry of the distribution around the median we also computed the skewness of the distribution of step length asymmetries associated with the lowest metabolic costs. Repeated measures ANOVAs were performed to test for differences in metabolic cost between the most and least costly asymmetries and symmetry during SplitFBK trials.

A linear mixed effects model was used to represent the relationship between metabolic cost and step length asymmetry. In this model we defined the independent variables to be: (1) asymmetry (*Asym*), which corresponds to the magnitude of the average step length asymmetry during each trial, (2) a categorical variable Leg with values of −1 when the slow leg took longer steps than the fast leg and 1 when the fast leg took longer steps, and (3) an interaction between *Leg* and *Asym* which was used to determine whether asymmetries in one direction were more costly than in the other. The model structure was defined as follows:

3 $$Met\,Power={\beta }_{0}+{\beta }_{1}Asym+{\beta }_{2}Leg+{\beta }_{3}Asym\ast Leg+{b}_{0}+{b}_{1}Asym+{b}_{2}Leg+{b}_{3}Asym\ast Leg$$

*B*_*i*_ (*i* = 0, 1, 2, 3) correspond to the coefficients for the fixed effects and *b*_*j*_(j = 0, 1, 2, 3) correspond to the coefficients for the random effects. These random effects were included to account for variability in metabolic power due to unexplained differences between subjects. A random slope was allowed for asymmetry, leg, and their interaction. Final model selection was achieved using a likelihood ratio test which compared the full model with simplified versions of the model (without random slopes and intercepts) to test the hypothesis that additional terms were necessary.

Linear mixed effects models were also used to test whether step length asymmetry induced systematic changes in stride time as previous research has shown that changes in cadence^8^ affect the metabolic cost of walking, and could, therefore, influence our results. We also fit linear mixed effects models to test whether changes in metabolic cost were associated with changes in stride time (Equations S1 and S2).

Next, to identify how differences in lower extremity mechanics, measured as the net braking and propulsive impulses, related to changes in asymmetry, we used the model shown in Equation 4. Here, the net impulse is expressed as the sum of the propulsive and braking impulse magnitudes, baseline corrected to the TiedFBK condition.

4 $${\rm{\Delta }}NetImpulse={\beta }_{0}+{\beta }_{1}Asym+{\beta }_{2}Leg+{\beta }_{3}Asym\ast Leg+{b}_{0}+{b}_{1}Asym+{b}_{2}Leg+{b}_{3}Asym\ast Leg$$

For the distribution of step length asymmetries that minimized the net impulse, defined as the sum of the magnitude of fast and slow braking and propulsive impulses, normality and skewness were tested as described for metabolic cost, and the median and interquartile range were identified to determine whether symmetry was associated with the mechanical optima.

Finally, to determine whether a more generalized estimate of effort combining each of our measures of energetic cost was optimized during adaptation, we derived z-scores for metabolic and mechanical metrics in our full dataset and for metabolic, mechanical and perceptual metrics in N = 15 participants. We summed these scores to obtain a composite estimate of cost. The distribution of asymmetries that minimized this composite metric was analyzed as described above. We also used the linear mixed effects model described in Eq. 5 to determine whether the composite cost was associated with asymmetry magnitude or direction. Given that the z-scores are standardized metrics for each participant, the inclusion of random effect terms was not necessary.

5 $$Composite\,Cost={b}_{0}+{b}_{1}Asym+{b}_{2}Leg+{b}_{3}Asym\ast Leg$$

For the PostFBK condition, we tested the hypothesis that the asymmetry participants selected at the end of this period would be associated with either (1) their natural, baseline asymmetry, (2) the asymmetry that minimized energetic costs, (3) or the asymmetry they experienced during their last trial. These options would be consistent with hypotheses that adaptation is driven by (1) error-minimization, (2) energetic optimization, and (3) recent experience, respectively. In 15 participants, a composite cost that included mechanical, metabolic and perceptual metrics of energetic cost was derived and used in the subsequent analysis. For this analysis, we fit linear mixed effects models for late-PostFBK of the form:

6 $$SLAPostFBK={\beta }_{0}+{\beta }_{1}SL{A}_{Baseline}+{\beta }_{2}SL{A}_{MinPower}+{\beta }_{3}SL{A}_{FinalSplitFBK}+{\beta }_{4}SL{A}_{minNetImpulse}+{\beta }_{5}SL{A}_{minComposite}+{b}_{0}+{b}_{1}SL{A}_{Baseline}+{b}_{2}SL{A}_{MinPower}+{b}_{3}SL{A}_{FinalSplitFBK}+{b}_{4}SL{A}_{minNetImpulse}+{b}_{5}SL{A}_{minComposite}$$

For this analysis, we defined the step length asymmetry during PostFBK as the average step length asymmetry during the last 10 strides of the trial, after participants had already adapted to the split belt, i.e., late adaptation^32, 53^. Therefore, the analysis of this portion of the experiment avoided the initial period where participants were potentially adjusting to the absence of visual feedback.

## Electronic supplementary material

Supplementary Materials
