# Supplementary material for: Evidence of Energetic Optimization during Adaptation Differs for Metabolic, Mechanical, and Perceptual Estimates of Energetic Cost
Source: Sci Rep. 2017 Aug 9;7:7682. doi: 10.1038/s41598-017-08147-y (PMC5550492; doi:10.1038/s41598-017-08147-y)
Supplement: Supplementary file 1 — Supplementary Materials [file 41598_2017_8147_MOESM1_ESM.pdf]

Supplementary Materials

Evidence of Energetic Optimization during Adaptation Differs for Metabolic, Mechanical, and Perceptual Estimates of Energetic Cost

Natalia Sánchez, Ph.D. [1], Sungwoo Park, M.S. [1], and James M. Finley, Ph.D.[1]
[1]Division of Biokinesiology and Physical Therapy, University of Southern California, Los Angeles, CA, 90033

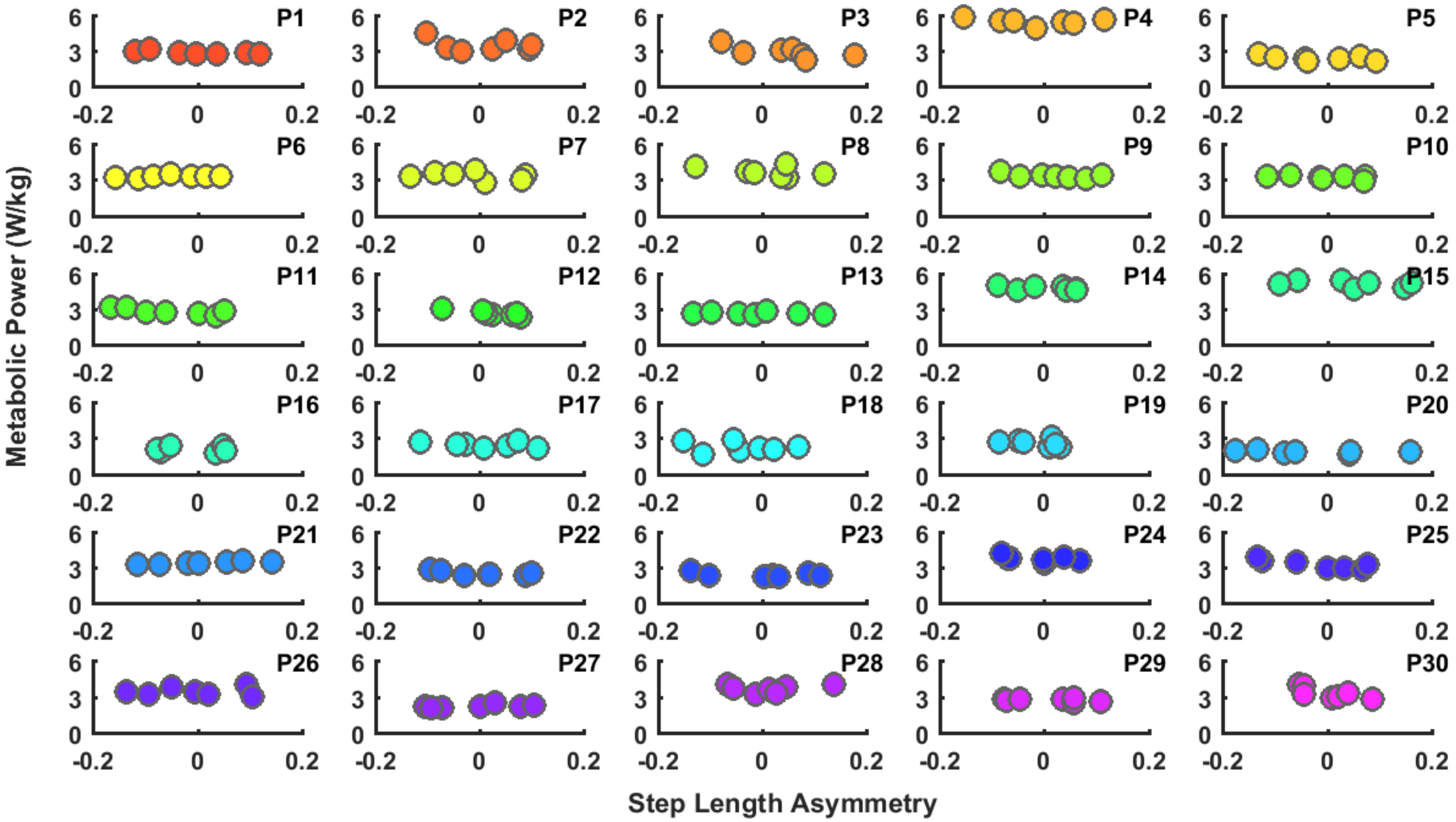

Figure S1. Individual metabolic power data for all participants P_i for i = 1,…,30, as a function of step length asymmetry.

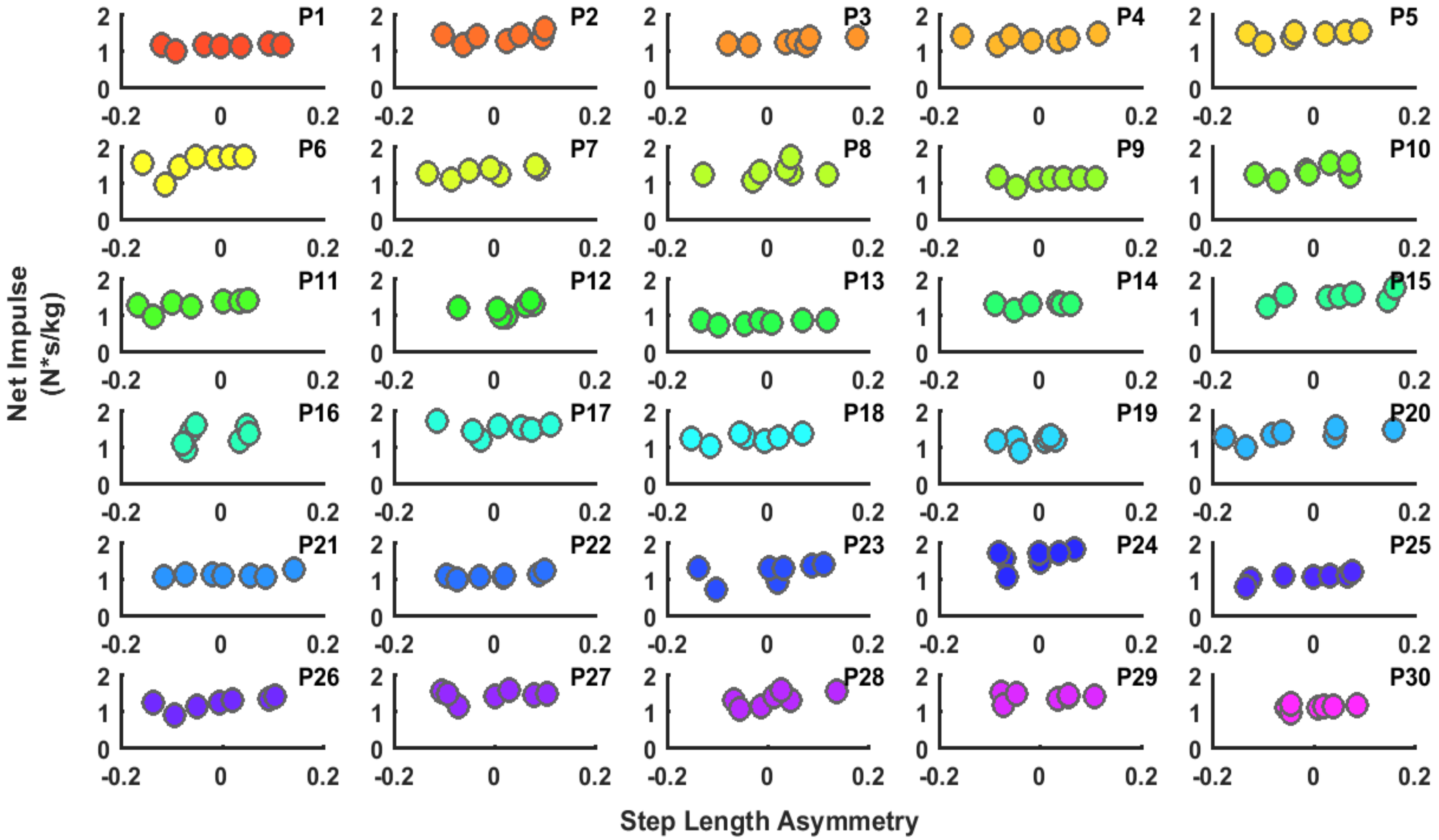

Figure S2. Individual net impulse data (sum of braking and propulsive impulse magnitude) for all participants P_i for i = 1,…,30, as a function of step length asymmetry.

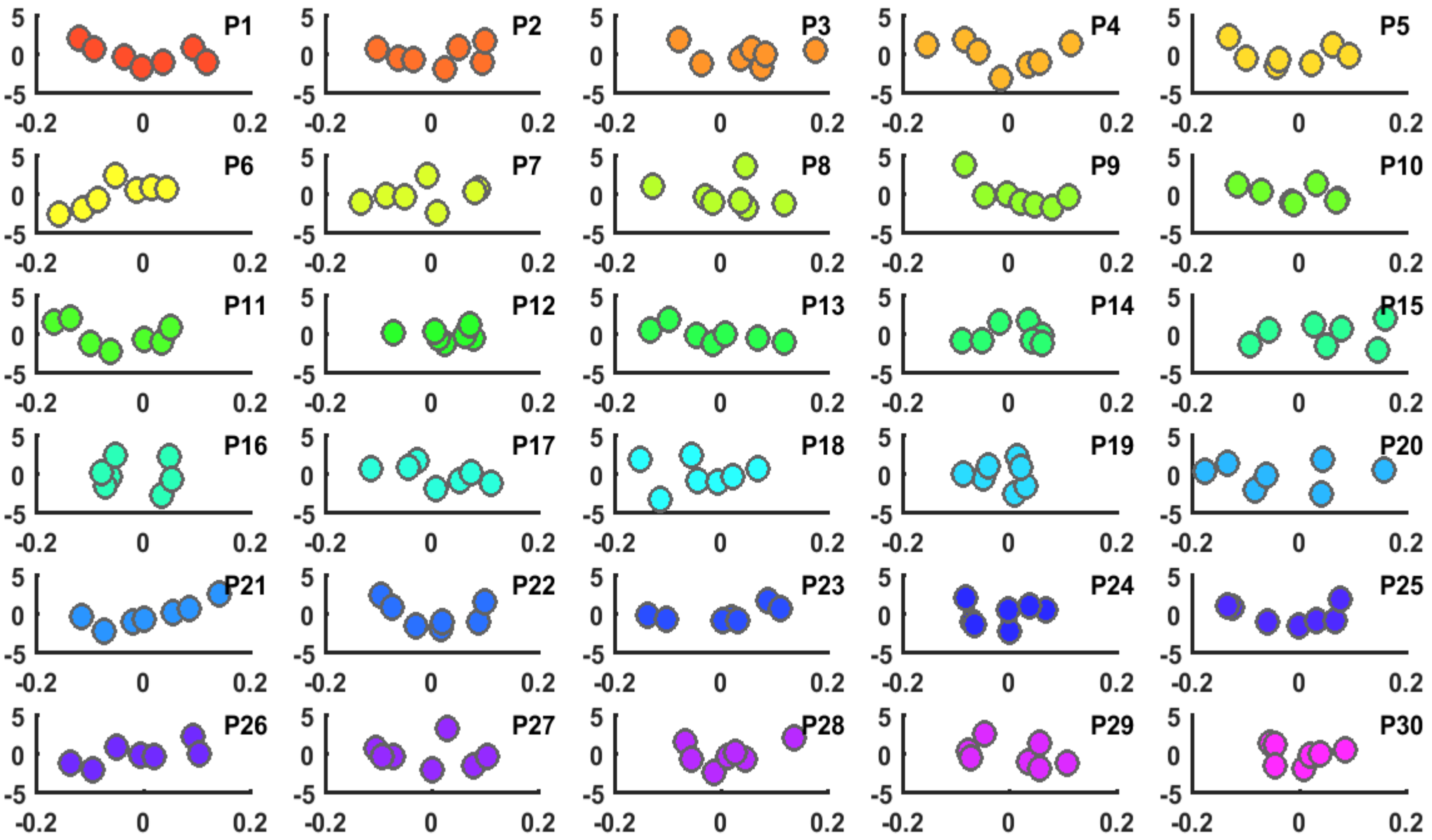

Figure S3. Individual composite energetic cost z-scores (for impulses and metabolic cost) for all participants P_i for i = 1,…,30, as a function of step length asymmetry.

Supplementary Experimental Procedures

Assessing Associations between Stride Time, Step Length Asymmetry, and Metabolic Cost

Mixed-effect models were used to determine if step length asymmetry induced systematic changes in stride time during the SplitFBK trials (Equation S1) and if variation in stride time influenced metabolic cost (Equation S2). The model was defined as follows, with SLA indicating step length asymmetry.

$$StrideTime\ = \beta_0 + \beta_1 SLA + b_0 + b_1 SLA \quad \text{(S1)}$$
$$MetPower\ = \beta_0 + \beta_1 StrideTime\ + b_0 + b_1 StrideTime \quad \text{(S2)}$$

Associations between lower extremity mechanics and metabolic power

We used the model shown in Equation S3 to explore the relationship between metabolic power and the braking and propulsive impulses for each limb with the goal of determining the extent to which changes in the mechanical demands of the task were associated with observed changes in metabolic power. Both metabolic power and impulses were expressed as the raw magnitude, without correcting for the effect of the visual feedback, to determine what percentage of the metabolic requirements is driven by the mechanics of walking. The magnitude of all impulses was summed to obtain the net impulse.

$$MetPower = \beta_0 + \beta_1 N\text{etImpulse} + b_0 + b_1 \text{NetImpulse} \quad \text{(S3)}$$

β_i (i = 0, 1) correspond to the coefficients for the fixed effects and b_j (j = 0, 1) correspond to the coefficients for the random effects. The random effect terms allow the model to assign an overall random intercept for each participant and a participant-specific random slope relating metabolic power and each impulse variable.

Effect of Trial History on Energetic Cost

We analyzed changes in metabolic and mechanical costs as a function of the change in step length asymmetry from the previous trial using the models defined in equations S4 and S5, to determine whether trial-to-trial changes in step length asymmetry influenced metabolic and mechanical cost (net impulses).

$$\Delta MetPower = \beta_0 + \beta_1 ChangeAsymm + b_0 + b_1 ChangeAsymm \quad \text{(S4)}$$
$$\Delta NetImpulse = \beta_0 + \beta_1 ChangeAsymm + b_0 + b_1 ChangeAsymm \quad \text{(S5)}$$

Test-Retest Reliability of Step Length Asymmetry and Metabolic Cost

Test-retest reliability was established in three participants who were tested on two separate days to determine whether our step length asymmetry and metabolic cost measurements were repeatable across multiple testing sessions. Participants were tested under BASELINE, TiedFBK and three SplitFBK conditions with target asymmetries of -0.10, 0 and 0.10. Reliability was quantified using the intra-class correlation coefficient derived from a two way mixed analyses to determine absolute agreement between sessions.

Supplementary Results

Effects of voluntary modification of foot placement on energetic cost

Table S1: Parameter values for model relating metabolic power to step length asymmetry

Fixed Effects	Estimate	Standard Error	t-Statistic	p-value
**Intercept**	0.677	0.122	5.526	p<0.001
**Leg**	-0.033	0.073	-0.453	0.651
**Asymmetry**	2.255	0.659	3.422	p<0.001
**Leg*Asymmetry**	-1.915	0.965	-1.986	0.048

Random Effects	Type	Estimate	Lower Limit	Upper Limit
**ID**	(std)	0.603	0.463	0.786

Changes in stride duration due to changes in step length asymmetry

There was a significant association between step length asymmetry and stride duration (Table S4). Specifically, stride duration increased for more positive values of step length asymmetry.

Table S2: Parameter values for model relating stride duration to step length asymmetry

Fixed Effects	Estimate	SE	t-Statistic	p-value
**Intercept**	1.253	0.0301	41.626	p<0.001
**SLA**	0.563	0.181	3.10	0.002

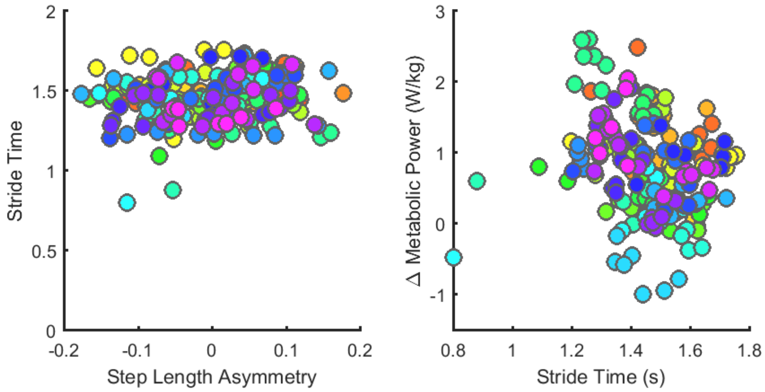

Figure S4. a) Stride time vs. achieved step length asymmetry for all participants and b) stride time vs. change in metabolic power for all participants. Participants (N = 30) are coded by color.

Changes in metabolic cost due to changes in stride duration

No significant main effects of stride time on metabolic cost were observed (Table S5). However, significant random effects in our model indicate variability in metabolic cost due to individual-specific changes in stride time. These difference across individuals are accounted for by the random intercept and slopes of the relationship between stride time and metabolic cost. These random effects are indicated in Table S5.

Table S3: Parameter values for model relating metabolic cost and stride time

Fixed Effects	Estimate	SE	t-Statistic	p-value
**Intercept**	1.07	0.248	4.316	p<0.001
**Stride Time**	-0.256	0.189	-1.359	0.175

Random Effects	Type	Estimate	Lower Limit	Upper Limit
**ID**	(std)	0.874	0.531	1.436
**StrideTime**	(corr)	-0.762	-0.931	-0.326
**StrideTime**	(std)	0.611	0.305	1.223

Associations between step length asymmetry and lower extremity mechanics

Table S4: Parameter values for model relating net impulses and step length asymmetry

Fixed Effects	Estimate	Standard Error	t-statistic	p-value
**Intercept**	0.057	0.023	2.477	0.014
**Leg**	-0.0011	0.013	-0.087	0.930
**Asymmetry**	0.355	0.166	2.129	0.034
**Leg*Asymmetry**	0.380	0.167	2.276	0.024

Random Effects	Type	Estimate	Lower Limit	Upper Limit
**ID**	(std)	0.107	0.081	0.143

Associations between lower extremity mechanics and metabolic power

Across asymmetries, there were systematic variations in the net braking and propulsive impulses for each limb (Fig S2). Results from the linear mixed effect model indicated that about 90% of the variability in metabolic power can be explained by the net impulse requirements (sum of braking and propulsive impulse magnitudes).

Table S5: Parameter values for model relating metabolic power to the net impulse

Fixed Effects	Estimate	Standard Error	t-statistic	p-value
**Intercept**	2.611	0.325	8.038	p<0.001
**NetImpulse**	0.463	0.217	2.177	0.030

Random Effects	Type	Estimate	Lower Limit	Upper Limit
**ID**	(std)	0.836	0.643	1.086

Associations between step length asymmetry and composite estimates of energetic cost

Table S6: Parameter values for model relating a composite cost score (mechanical + metabolic) to step length asymmetry

Fixed Effects	Estimate	Standard Error	t-Statistic	p-value
**Intercept**	-0.535	0.172	-3.103	0.002
**Asymmetry**	8.289	2.251	3.681	p<0.001

Table S7: Parameter values for model relating a composite cost score (mechanical + metabolic + perceptual) to step length asymmetry

Fixed Effects	Estimate	Standard Error	t-Statistic	p-value
**Intercept**	-0.629	0.295	-2.131	0.036
**Asymmetry**	10.044	3.956	2.539	0.013

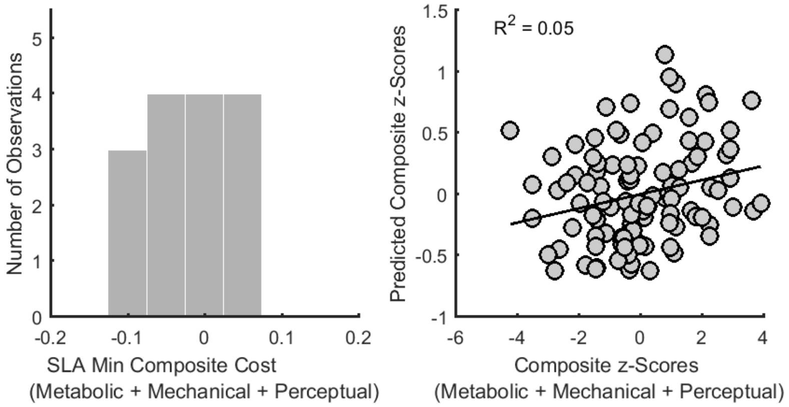

Figure S5. Associations between the composite energetic cost using all three metrics of energetics and step length asymmetry. a) Histogram of step length asymmetries associated with the minimum composite cost. b) Plot of measured vs. predicted composite costs. Adjusted R^2=0.05.

Effect of Trial History on Energetic Cost

There was a significant main effect of the trial-to-trial change in step length asymmetry on metabolic cost and net impulse. The trial by trial change in step length asymmetry explained 14% of the variability in the increase in metabolic cost (from TiedFBK) and 44% of the variability in net impulse, Fig S6.

Table S8: Parameter values for model relating trial by trial change in metabolic cost and asymmetry

Fixed Effects	Estimate	SE	t-Statistic	p-value
**ChangeAsymm**	1.348	0.241	-5.587	p<0.001

Table S9: Parameter values for model relating trial by trial change in net impulse and asymmetry

Fixed Effects	Estimate	SE	t-Statistic	p-value
**Intercept**	-0.018	0.008	-2.036	0.043
**ChangeAsymm**	0.940	0.077	12.087	p<0.001

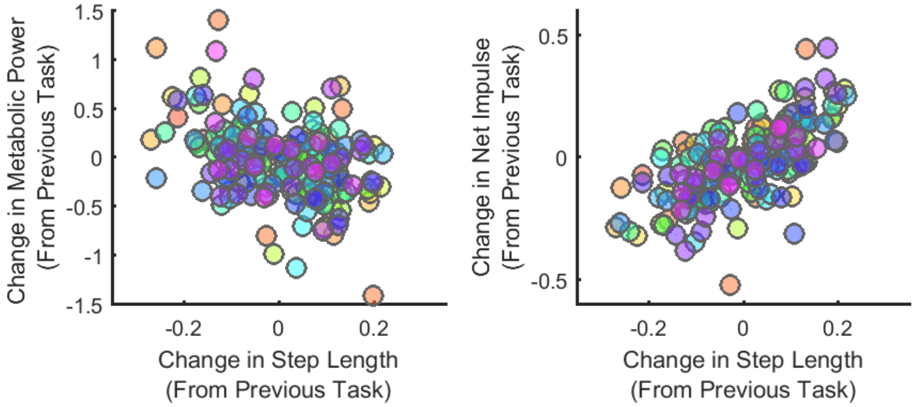

Figure S6. Trial by trial change in metabolic cost and net impulses (both measured as the change from TiedFBK conditions).

Test-Retest Reliability

For step length asymmetry we obtained an intra-class correlation of 0.97 between day 1 and day 2. The intra-class correlation for measurements of metabolic cost (in W/kg) on day 1 vs day 2 was 0.967 ($p<0.001$). Results demonstrates that day-to-day behavior was highly reliable for individual participants.

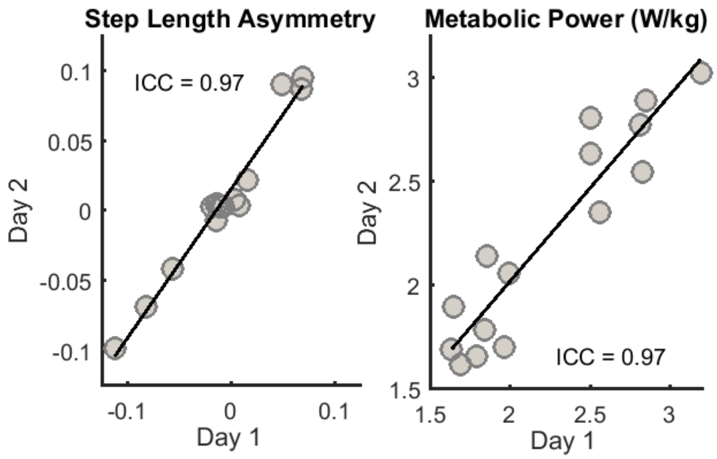

Figure S7. Measurements for step length asymmetry and metabolic cost on day 1 and 2 for assessment of test-retest reliability.
